# HSPA12B Attenuated Acute Myocardial Ischemia/reperfusion Injury via Maintaining Endothelial Integrity in a PI3K/Akt/mTOR-dependent Mechanism

**DOI:** 10.1038/srep33636

**Published:** 2016-09-20

**Authors:** Qiuyue Kong, Leyang Dai, Yana Wang, Xiaojin Zhang, Chuanfu Li, Surong Jiang, Yuehua Li, Zhengnian Ding, Li Liu

**Affiliations:** 1 Department of Anesthesiology, First Affiliated Hospital with Nanjing Medical University, Nanjing 210029, China; 2 Department of Geriatrics, First Affiliated Hospital with Nanjing Medical University, Nanjing 210029, China; 3 Departments of Surgery, East Tennessee State University, Johnson City, TN37614, USA; 4 Department of Pathophysiology, Nanjing Medical University, Nanjing 210029, China.

## Abstract

Endothelial damage is a critical mediator of myocardial ischemia/reperfusion (I/R) injury. HSPA12B is an endothelial-cell-specifically expressed heat shock protein. However, the roles of HSPA12B in acute myocardial I/R injury is unknown. Here we reported that myocardial I/R upregulated HSPA12B expression in ventricular tissues, and endothelial overexpression of HSPA12B in transgenic mice (Tg) limited infarct size, attenuated cardiac dysfunction and improved cardiomyocyte survival compared with their wild type littermates. These improvements were accompanied with the diminished myocardial no-reflow phenomenon, decreased microvascular leakage, and better maintained endothelial tight junctions. The I/R-evoked neutrophil infiltration was also suppressed in Tg hearts compared with its wild type (WT) littermates. Moreover, Tg hearts exhibited the enhanced activation of PI3K/Akt//mTOR signaling following I/R challenge. However, pharmacological inhibition of PI3K abolished the HSPA12B-induced cardioprotection against myocardial I/R injury. The data demonstrate for the first time that the endothelial HSPA12B protected hearts against myocardial I/R injury. This cardioprotective action of HSPA12B was mediated, at least in part, by improving endothelial integrity in a PI3K/Akt/mTOR-dependent mechanism. Our study suggests that targeting endothelial HSPA12B could be an alternative approach for the management of patients with myocardial I/R injury.

Timely restoration of blood flow (reperfusion) to the ischemic myocardium is the standard treatment for the patients with myocardial ischemia. Reperfusion after ischemia is demonstrated to limit infarct size, improve long-term myocardial function, and more importantly, reduce mortality^1^. However, reperfusion can induce additional damage to myocardium, known as ischemia/reperfusion (I/R) injury which is manifest as aggravated functional impairment, accelerated cardiomyocyte death and arrhythmia^1^,^2^. Management of I/R injury, therefore, is important for improving the outcome of these patients.

The development of myocardial I/R injury involves multiple mechanisms. Among of them, endothelial cell (EC) damage has been shown to be a critical mediator. Endothelial cells (ECs) comprise the inner layer of arteries, veins, endocardial epithelium, and myocardial capillaries^3^,^4^. While ECs are less sensitive to ischemia, they are quite sensitive to I/R in the heart compared with cardiomyocytes^3^. Evidence has demonstrated that I/R upregulates expression of surface adhesion molecules on ECs and recruits neutrophils, where by not only further damages ECs but also leads to neutrophils migrating into interstitial myocardium. Moreover, I/R increases endothelial permeability by disruption of endothelial barrier function, which will aggravate inflammation and no-reflow phenomenon in myocardium following I/R^5^. Therefore, maintaining ECs integrity serves as a promising therapeutic target for the treatment of myocardial I/R injury.

Heat shock protein A12B (HSPA12B) is a new member of heat shock protein superfamily^6^,^7^. In different with the ubiquitous expression of other heat shock proteins, HSPA12B expresses specifically in ECs^6^,^7^,^8^. This unique distribution pattern suggests a possible involvement of HSPA12B in the endothelial-related pathophysiological events. Indeed, HSPA12B has been shown to be essential for endothelial proliferation and migration^6^,^7^. Moreover, we have reported that HSPA12B suppressed the endothelial inflammatory responses induced by endotoxin^9^,^10^. These observations suggest a functional role of HSPA12B in maintaining endothelial homeostasis. However, the role of HSPA12B in acute myocardial I/R injury is not clear.

To answer this question, we examined the effects of HSPA12B on myocardial I/R injury at acute phase in mice. We observed that the I/R-induced myocardial injury was attenuated by HSPA12B overexpression. This action of HSPA12B was mediated, at least in part, by improving endothelial integrity in a PI3K/Akt/mTOR-dependent mechanism. Our study suggests that targeting HSPA12B could be an alternative approach for the management of patients with myocardial I/R injury.

## Results

### Myocardial I/R increases HSPA12B expression

**Figure 1:**
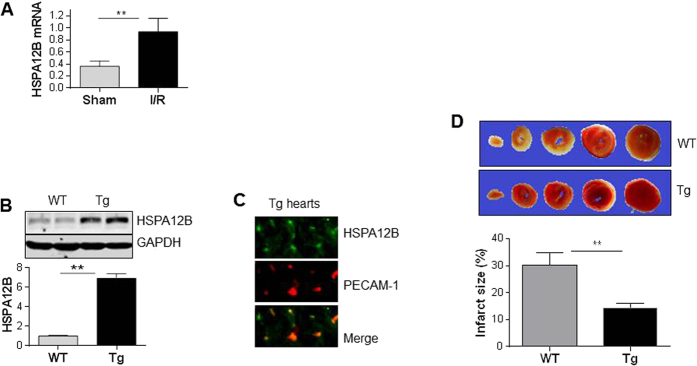
HSPA12B overexpression in endothelial cells limited infarct size following myocardial I/R. (**A**) Upregulation of HSPA12B by I/R. Ventricular tissues were collected from WT mice 4 h after I/R. Total RNA was extracted for examination of HSPA12B mRNA levels. The mRNA levels of β-actin served as internal controls. **P < 0.01, n = 6 per group. (**B**) Overexpression of HSPA12B in Tg hearts. Ventricular tissues were collected from 8-week old mice. Cellular extracts were prepared for immunoblotting against HSPA12B. The blots against GAPDH served as loading controls. **P < 0.01, n = 4 per group. (**C**) Colocalization of HSPA12B with PECAM-1 in Tg hearts. Ventricular tissues were collected from HSPA12B Tg mice aged of 8-week old. Cryosectioning was prepared for the immunofluorescence staining against HSPA12B and PECAM-1, a selective marker of endothelial cells. Note that the staining of HSPA12B (green) was colocalized with PECAM-1 (red). The representative images were from three independent mice. (**D**) Infarct size. TTC staining was performed to analyzed infarct size (pale white) 4 h after I/R. **P < 0.01, n = 5 per group.

To determine the possible involvement of HSPA12B in acute myocardial I/R injury, we examined HSPA12B expression 4 h after I/R in C57/BL6 mice. The HSPA12B mRNA levels were increased by 157.8% in ventricular tissues following I/R compared with sham controls (*P* < 0.01) (Fig. 1A).

### Endothelial overexpression of HSPA12B limits infarct size induced by I/R

To investigate the direct roles of upregulated HSPA12B in myocardial I/R injury, we generated transgenic mice (Tg) with overexpression of HSPA12B in endothelial cells. The successful transgene was confirmed by immunoblotting analysis, which demonstrated a greater HSPA12B expression in Tg hearts compared with its wild type (WT) littermates (*P* < 0.01, Fig. 1B). While in Tg hearts, the expression of HSPA12B was colocalized with PECAM-1, a selective marker of endothelial cells (Fig. 1C). Collectively, the data suggest an overexpression of HSPA12B in myocardial endothelial cells of Tg mice.

We then examined the effects of HSPA12B on I/R-induced myocardial infarction. As shown in Fig. 1D, the infarct sizes were 30.3% and 14.4% in WT and Tg hearts after I/R, respectively. Therefore, Tg hearts exhibited a significant smaller size (52.3%) of infarcts than WT hearts (*P* < 0.01). The areas at risk reflected by Evan blue perfusion were comparable between WT and Tg hearts (Figure S1).

### HSPA12B attenuates cardiac dysfunction induced by I/R

**Figure 2:**
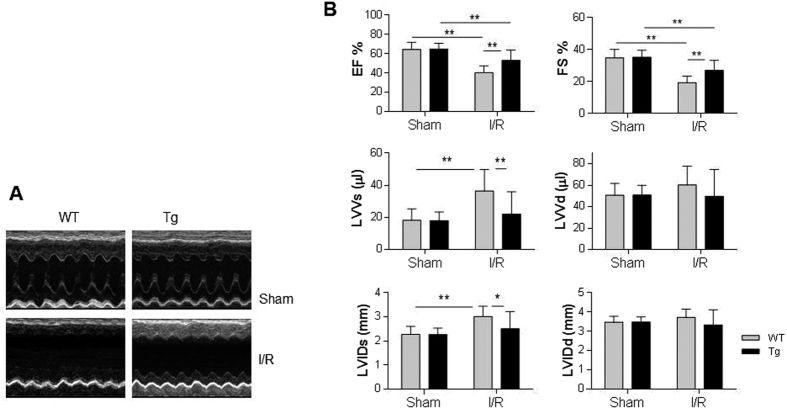
Overexpression of HSPA12B attenuated the I/R-induced cardiac dysfunction. (**A**) M-mode tracings of echocardiography. Cardiac function was examined using two-dimentional echocardiography 24 h after I/R. M-mode tracings were recorded at papillary levels of left ventricles. The representative images were from 10–17 mice in each group. (**B**) Parameters of cardiac dysfunction. Parameters of cardiac function were measured digitally on the M-mode tracings and averaged from three to five cardiac cycles. **P < 0.01 and *P < 0.05, n = 10–17 per group.

Echocardiography was used to evaluate cardiac function 24 h after I/R. As shown in Fig. 2A,B, I/R significantly decreased ejection fraction (EF%, 37.5%) and fraction shortening (FS%, 44.3%), while increased left ventricular volume and internal diameter at systolic phases (LVVs and LVIDs: 18.7% and 99.6%, respectively) in WT mice, compared with WT sham controls (*P* < 0.01). By striking contrast, the I/R-induced decreases of EF% and FS%, and increases of LVIDs and LVVs were attenuated significantly in Tg mice by 31.7%, 39.2%, 17.5% and 39.4%, respectively, compared with WT mice (*P* < 0.01 or 0.05). Left ventricular volume and internal diameter at diastolic phases (LVVd and LVIDd) showed no significant changes between WT and Tg mice in both sham and I/R groups, suggesting the myocardial compliance was still compensated. No significant difference of cardiac function was detected between sham WT and Tg mice. The data suggest that HSPA12B overexpression improved cardiac function following I/R.

### HSPA12B increases cardiomyocyte survival following I/R

**Figure 3:**
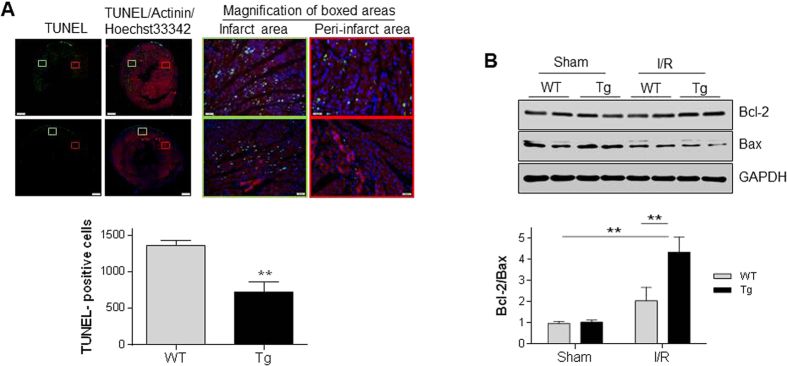
HSPA12B overexpression reduced cardiomyocyte apoptosis following I/R. (**A**) Apoptosis assay. Ventricular tissues were collected 4 h after I/R for paraffin-embedded sectioning. A TUNEL assay was performed to detect cardiomyocyte apoptosis (green). Alpha-actinin was used to stain cardiomyocytes (red). Hoechst 33342 reagent was used to counterstain the nuclei (blue). TUNEL-positive cardiomyocytes were observed using a fluorescent microscope. The magnification of boxed areas was shown in the right-hand panel. Total cardiomyocyte apoptosis was counted in each slide. **P < 0.01, n = 4 per group. (**B**) Bcl-2/Bax ratios. Ventricular tissues were collected 4 h after I/R. Cellular extracts were prepared for immunoblotting against Bcl-2 and Bax. The densities of each bands were quantified and the ratios of Bcl-2/Bax were calculated. The blots against GAPDH served as loading controls. **P < 0.01, n = 4 per group.

Apoptosis in cardiomyocytes plays an important role in the development of myocardial I/R injury^11^. As shown in Fig. 3A, I/R increased apoptotic cardiomyocytes to 1362.3 and 720.5 per slide view of WT and Tg hearts, respectively. Barely cardiomyocyte apoptosis was detected in both sham groups as demonstrated in our previous studies^12^. Thus, the I/R-induced cardiomyocyte apoptosis was attenuated by 47.1% in Tg hearts compared with WT controls (*P* < 0.01). Consistently, the anti-apoptotic Bcl-2/Bax ratios were significantly increased in Tg hearts by 103.1% compared with WT hearts following I/R (*P* < 0.01) (Fig. 3B).

### HSPA12B diminishes microvascular no-reflow phenomenon after reperfusion

**Figure 4:**
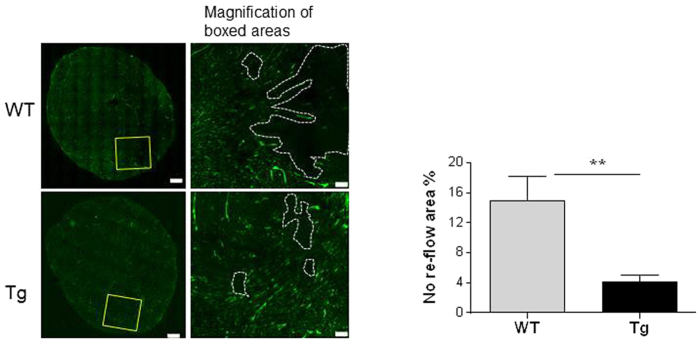
HSPA12B overexpression attenuated myocardial microvascular no-reflow phenomenon after reperfusion. The myocardial microvascular reflow was detected 30 min after I/R using FITC-labeled *Lycopersicon esculentum* (Tomato) lectin. The magnification of boxed areas were illustrated in the right-handed panels. Note that the I/R-induced no flow (areas within dotted lines) was diminished in HSPA12B Tg hearts during reperfusion phase. **P < 0.01, n = 4 per group.

The no-reflow phenomenon after reperfusion is a basic mechanism of myocardial I/R injury^4^,^13^. As shown in Fig. 4, a decreased microvascular perfusion was observed in WT hearts after reperfusion. By striking contrast, the I/R-induced no-reflow was prominently diminished in Tg hearts. The no-reflow areas were significant smaller in Tg hearts than that in WT hearts after reperfusion (4.2 ± 0.9% vs. 14.9 ± 3.3%, *P* < 0.01).

### HSPA12B attenuates microvascular leakage following I/R

**Figure 5:**
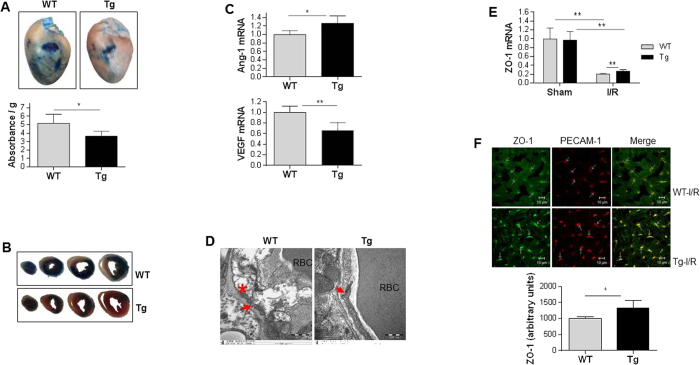
HSPA12B overexpression improved endothelial integrity following I/R. (**A**)Vascular leakage. Vascular leakage in myocardium was measured by Evan’s blue extravasation at 30 min after *myo*cardial I/R. The data are expressed as absorbent units per gram ventricular tissues (absorbance/g). *P < 0.05, n = 5 per group. (**B**) Images of sectioned hearts from Evans blue extravasation. After photographing of whole hearts, the Evans blue perfused hearts were sectioned into 4 pieces with equal thickness. n = 5 per group. (**C**) Levels of VEGF and Ang-1 mRNA. Ventricular tissues were collected 4 h after I/R. Total RNA was extracted for the analysis of mRNA levels of VEGF and Ang-1 by real-time PCR. The mRNA levels of β-actin served as internal controls. **P < 0.01 and *P < 0.05, n = 4–5 per group. (**D**) EC-EC gap formation and ECs damages. Ventricular tissues were collected 4 h after I/R. Ultra-thin sections were prepared, stained and observed using a transmission electron microscope. Note that I/R induced EC-EC gap formation (→) and endothelial damages (*) in WT hearts, which was attenuated in Tg hearts (→ indicates the intact junction). The representative images were from three independent experiments. RBC: red blood cell. (**E**) Levels of ZO-1 mRNA. Ventricular tissues were collected 4 h after I/R. Total RNA was extracted for the analysis of mRNA levels of ZO-1. The mRNA levels of β-actin served as internal controls. **P < 0.01, n = 3–5 per group. (**F**) ZO-1 immunofluorescence in ECs. Ventricular tissues were collected 24 h after I/R. Cryosectioning was prepared and co-stained with ZO-1 (green) and PECAM-1 (red), a selective marker of endothelial cells. Note that HSPA12B Tg hearts demonstrated significant stronger fluorescence than WT hearts following I/R. **P < 0.01, n = 4 per group.

The disruption of microvascular barrier function contributes myocardial I/R injury^4^. We then examined microvascular permeability by Evans blue dye extravasation after I/R. As shown in Fig. 5A, the Evans blue dye extravasation was 5.2 and 3.6 absorbance/gram ventricular tissues in WT and Tg heart following I/R, respectively. Therefore, the I/R-provoked microvascular permeability was attenuated by 29.5% in Tg heart compared with WT controls (*P* < 0.05). The Evans blue leakage in myocardium was shown in transverse sections (Fig. 5B). In consistent with this, Tg hearts exhibited significant higher levels of Angiopoetin-1 (anti-permeability) by 26.7% and lower levels of VEGF (promoting permeability) expression by 33.7%, respectively, compared with WT controls after I/R (*P* < 0.01 or 0.05) (Fig. 5C).

### HSPA12B maintains the intact of EC-EC tight junction and prevents ECs damages after I/R

The intact of EC-EC tight junctions and detachment of ECs from basement membrane were evaluated using the transmission electron microscope according to previous methods^4^. An obvious EC-EC gap formation, ECs damage and detachment were detected in WT hearts following I/R (Fig. 5D). However, the I/R-induced these abnormalities were attenuated in Tg hearts. Tg hearts showed more intact EC-EC junctions and less EC damage than WT hearts after I/R.

ZO-1 plays important roles in the maintenance of EC-EC tight junction^14^. As shown in Fig. 5E, ZO-1 mRNA levels in WT hearts were downregulated (79.3%) following I/R compared with sham WT controls (*P* < 0.01). I/R also downregulated ZO-1 mRNA levels in Tg hearts (*P* < 0.01). However, Tg hearts exhibited significant higher levels of ZO-1 mRNA compared with WT hearts after I/R (*P* < 0.01). Consistent with this, immunofluorescence staining revealed a significant stronger staining of ZO-1 in PECAM-positive cells of Tg hearts by 32.4% compared with WT hearts following I/R (Fig. 5F), suggesting that HSPA12B maintained ZO-1 expression in ECs following I/R.

### HSPA12B reduces neutrophil infiltration and VCAM-1 induction following I/R

**Figure 6:**
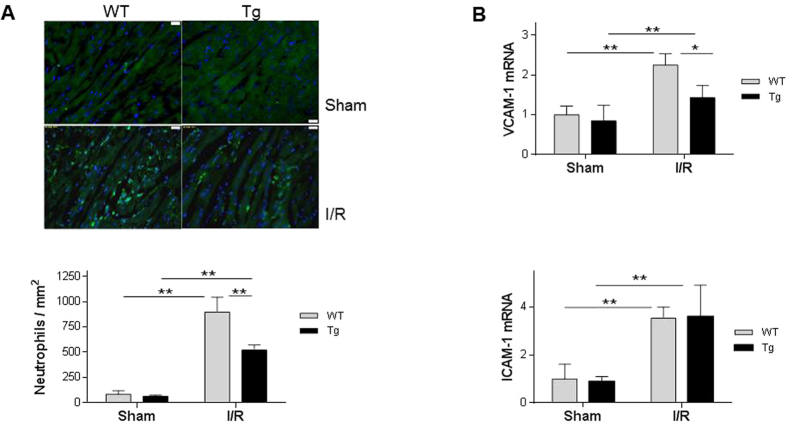
HSPA12B overexpression suppressed neutrophil infiltration following myocardial I/R. (**A**) Neutrophils infiltration. Ventricular tissues were collected 24 h after I/R. Cryosectioning was prepared for the immunofluorescence staining against neutrophils. Hoechst 33342 reagent was used to counterstain the nuclei. More than 16 fields on each slide were randomly examined. **P < 0.01, n = 4 per I/R group and n = 3 per sham group. (**B**) Levels of VCAM-1 and ICAM-1 mRNA. Ventricular tissues were collected 4 h after I/R. Total RNA was extracted for the analysis of mRNA levels of VACM-1 and ICAM-1 by real-time PCR. The mRNA levels of β-actin served as internal controls. **P < 0.01 and *P < 0.05, n = 4–6 per group.

Neutrophils trapping and accumulation in myocardium are key events for myocardial I/R injury^15^,^16^. We therefore examined neutrophil infiltration 24 h after I/R, because a maximal content of neutrophils in myocardium was observed at this time point^17^. As shown in Fig. 6A, I/R increased neutrophil infiltration by 9.6- and 6.9-fold in WT and Tg hearts, respectively, compared with their sham controls (*P* < 0.01). Therefore, the I/R-induced neutrophil infiltration was attenuated significantly by 41.8% in Tg hearts compared with WT controls (*P* < 0.01).

Adhesive molecules, including vascular cell adhesion molecule-1 (VCAM-1) and intercellular adhesion molecule-1 (ICAM-1), are critical for mediating the neutrophil trans-endothelial migration^18^. VCAM-1 mRNA levels were upregulated by 125.1% and 68.6% in WT and Tg hearts following I/R, respectively, compared with their sham controls (*P* < 0.01) (Fig. 6B). Thus, the I/R-induced VCAM-1 upregulation was reduced by 36.6% in Tg hearts compared with WT hearts (*P* < 0.05). ICAM-1 mRNA levels showed no significant difference between WT and Tg hearts after I/R.

### HSPA12B activates PI3K/Akt/mTOR signaling in I/R-challenged hearts

**Figure 7:**
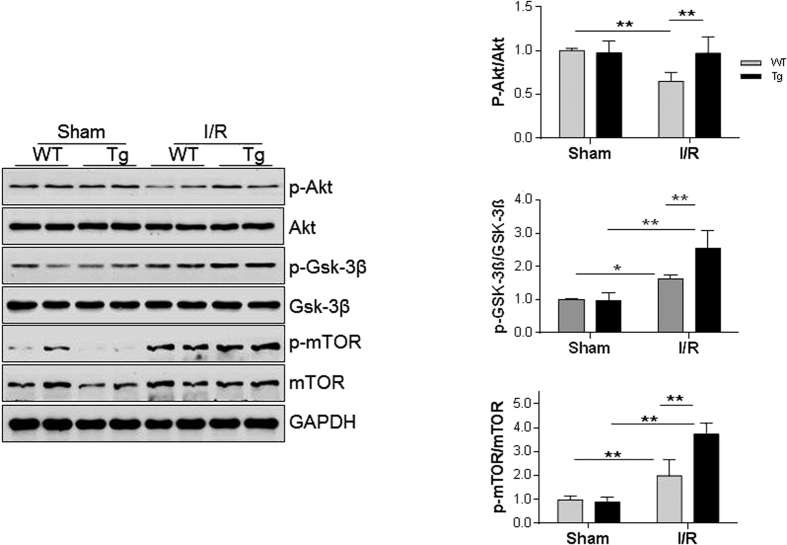
HSPA12B overexpression activated PI3K/Akt/mTOR signaling following myocardial I/R. Ventricular tissues were collected 4 h after I/R. Cellular extracts were prepared for immunoblotting against the indicated primary antibodies. The blots against GAPDH served as loading controls. **P < 0.01 and *P < 0.05, n = 4 per group. p-Akt: phosphor-Akt; p-GSK-3β: phosphor–GSK-3β; p-mTOR, phosphor-mTOR.

Activation of PI3K/Akt/mTOR signaling has been shown playing cardioprotective roles against I/R injury^19^,^20^,^21^. As shown in Fig. 7, I/R decreased the ratios of phosphor-Akt/Akt by 35.0% in WT hearts compared with sham WT controls (*P* < 0.05). By striking contrast, the ratios of phosphor-Akt/Akt were maintained at normal levels in Tg hearts following I/R in comparison with sham Tg controls. When compared with I/R-challenged WT hearts, phosphor-Akt/Akt ratios were significantly higher (49.2%) in I/R-challenged Tg hearts (*P* < 0.01).

GSK-3β and mTOR are important downstream targets of Akt. I/R increased the ratios of phosphor-GSK-3β/GSK-3β and phosphor-mTOR/mTOR by 62.6% and 101.3% in WT hearts, respectively, compared with the sham WT controls. Interestingly, the I/R-induced increases of phosphor-GSK-3β/GSK-3β and phosphor-mTOR/mTOR ratios were enhanced significantly by 56.9% and 88.3% in Tg hearts, respectively, compared with WT hearts (*P* < 0.01).

In sham groups, no significant differences of the ratios of phosphor-Akt/Akt, phosphor-Gsk-3β/Gsk-3β and phosphor-mTOR/mTOR were detected between WT and Tg hearts, though p-mTOR and mTOR levels were significantly downregulated in Tg hearts compared with WT controls.

### PI3K inhibition with Wortmannin abolishes the protective effects of HSPA12B on cardiac dysfunction following I/R

**Figure 8:**
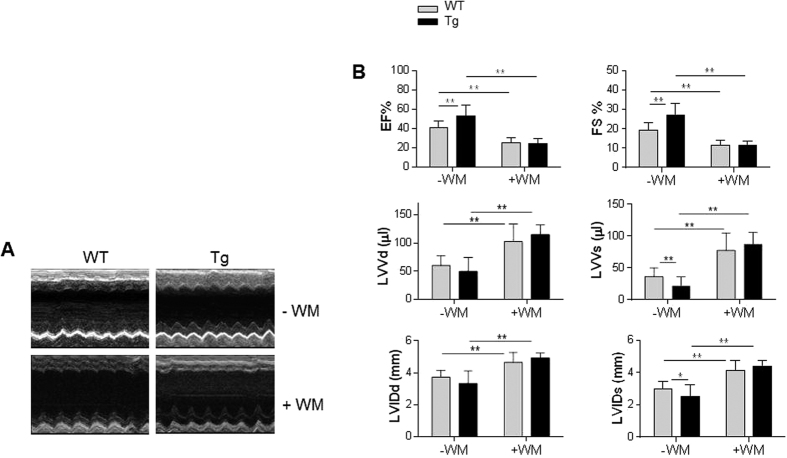
Inhibition of PI3K with Wortmannin abolished the HSPA12B-induced cardioprotection following myocardial I/R. Mice were administrated with Wortmannin (WM) at a dose of 1 mg/kg body weight and a volume of 8 μl/g body weight 60 min prior to ischemic insult. Cardiac function was examined using two-dimensional echocardiography 24 h after I/R. M-mode tracings were recorded at papillary levels of left ventricles (**A**). Parameters of cardiac function were measured digitally on the M-mode tracings and averaged from three to five cardiac cycles (**B**). **P < 0.01, n = 6–17 per group.

To determine the roles of PI3K/Akt/mTOR activation in the cardioprotection of HSPA12B against I/R, we administrated mice with Wortmannin, a widely used PI3K inhibitor^22^,^23^, 60 min prior to ischemic insult. Cardiac function was evaluated 24 h after I/R. Wortmannin preadministration significantly aggravated the I/R-induced cardiac dysfunction, as reflected by decreases in EF% and FS%, increases in LVVd, LVVs, LVIDd and LVIDs in both WT and Tg mice, respectively, compared with the I/R-challenged WT and Tg mice that did not receive Wortmannin treatment (*P* < 0.01). Most important, Tg mice exhibited no significant difference in cardiac dysfunction compared with WT mice after I/R in the presence of Wortmannin, suggesting that PI3K inhibition abolished the HSPA12B-induced cardioprotection against myocardial I/R injury (Fig. 8A,B).

## Discussion

The significant finding of this study is that HSPA12B Tg mice exhibited a decrease of infarct size and attenuation of cardiac dysfunction following myocardial I/R. Also, the I/R-provoked cardiomyocyte apoptosis, no-reflow phenomenon, neutrophils infiltration, vascular leakage and endothelial damages were attenuated in the hearts of Tg mice. Moreover, Tg hearts showed enhanced activation of PI3K/Akt/mTOR signaling compared following I/R, while inhibition of this signaling with Wortmannin abolished the cardioprotection of HSPA12B against I/R injury. Our data suggests that targeting HSPA12B could be an alternative therapeutic approach for the treatment of myocardial I/R injury.

We have reported previously that HSPA12B attenuated cardiac remodeling at the chronic phase of permanent ischemia without reperfusion via an eNOS-mediated angiogenesis mechanism^12^. In this study we examined the effect of HSPA12B on acute myocardial ischemia/reperfusion injury, because I/R possesses unique pathological process compared with permanent ischemia without reperfusion. Evidence demonstrates that the same molecule or biological event may exert different or even opposite effects on myocardial ischemia with or without reperfusion. For example, inhibition of GSK-3β attenuates I/R injury but exacerbates ischemic injury^24^. Similarly, autophagy alleviates energy crisis during myocardial ischemia whereas shows detrimental effects during reperfusion^25^. Fortunately, current study also demonstrated a protection of HSPA12B against myocardial I/R injury but the mechanism was different from the protection of HSPA12B against permanent ischemia.

The “no-reflow” is defined as incomplete and non-uniform reperfusion at the microvascular level despite adequate re-opening of the proximal artery after a period of transient ischemia^13^. As early as in 1960s, the no-reflow phenomenon after myocardial I/R has been demonstrated by experimental animal studies^26^. Also, no-reflow has been reported to occur in up to 60% of ST-segment elevation myocardial infarction patients with optimal coronary vessel reperfusion^27^. The no-reflow appears to persist at least four weeks, and has reproducibly been shown to significantly predict left ventricular segmental wall motion dysfunction, ventricular remodeling and adverse clinical outcome^28^. Therefore, diminishing “no-reflow” phenomenon or in another word, improving microvascular blood flow, serves as a promising approach for treating myocardial ischemia/reperfusion injury. In this study, we observed that the limited infract size and improved cardiac function in HSPA12B Tg mice were accompanied with a diminished microvascular no-reflow phenomenon after I/R, suggesting that HSPA12B promoted the restoration of microvascular blood flow after I/R.

The pathophysiological mechanisms responsible for the development of no-reflow after I/R have not been fully understood. However, endothelial damage, leukocyte plugging, microvascular obstruction, and mechanical compression by prominent intracellular and interstitial edema have been proposed as contributors for I/R-induced no-reflow^13^,^27^,^29^. Indeed, we observed that the I/R-induced neutrophil infiltration, EC-EC gap formation, EC detachment, and microvascular leakage were attenuated in HSPA12B Tg mice, suggesting that HSPA12B improved microvascular blood flow through multiple ways.

Neutrophils infiltration and trapping in interstitial myocardium have been detected in both clinical patients and experimental animals following I/R. Beside inducing no-reflow phenomenon, neutrophils exacerbates myocardial damage through releasing a large amount of ROS, upregulating proinflammatory cytokines, disrupting endothelial tight junction, and resulting in more neutrophils infiltration^4^,^15^. Inhibition of neutrophil infiltration has been shown protecting against myocardial I/R injury^16^,^30^. The adhesion of neutrophils to ECs is a critical step for the neutrophil migration and infiltration, while upregulation of adhesion molecules is critical for the neutrophil-EC binding^18^. Indeed, we observed that the I/R-induced neutrophil infiltration and VCAM-1 upregulation were attenuated in HSPA12B Tg heart. Our data suggest that inhibition of neutrophil infiltration contributed to the cardioprotection of HSPA12B against myocardial I/R injury.

Disruption of endothelial integrity is a common pathological process following myocardial I/R, which contributes to vascular leakage and neutrophil infiltration and ultimately results in cardiomyocyte apoptosis and impairs myocardial function^3^,^5^. We observed that the myocardial I/R-provoked endothelial damages and EC-EC gap formation were significantly attenuated in HSPA12B Tg hearts in this study. Moreover, the myocardial microvascular hyperpermeability were reduced in Tg hearts following I/R. Additionally, the HSPA12B Tg hearts exhibited a prominent improvement of no-reflow during reperfusion after ischemia. These data indicate a better maintained endothelial integrity in HSPA12B overexpressed hearts following I/R. In supporting these results, HSPA12B has been shown to protect endothelial barrier function in endothelial monolayers, lungs and hearts during endotoxemia by our previous studies^8^,^9^,^31^. The integrity of endothelial barrier depends upon intercellular junctional complex between adjacent ECs, while ZO-1 has been shown as one of the critical components in this complex^14^. We observed that the I/R-induced downregulation of ZO-1 mRNA was attenuated in HSPA12B Tg hearts. More interestingly, Tg hearts demonstrated stronger ZO-1 immunofluorescence in PECAM-1-positive cells following I/R. These data suggest that HSPA12B increased ZO-1 expression in myocardial endothelial cells following I/R, which may contribute to improve the endothelial integrity. Taken together, our data suggest that HSPA12B maintained endothelial integrity which in turn contributed to the cardioprotection from myocardial I/R injury.

Activation of PI3K/Akt/mTOR signaling has been well demonstrated in the protection against myocardial I/R injury^19^,^24^. The mechanisms responsible for the cardioprotection of this signaling include increasing cardiomyocyte survival, reducing neutrophil and macrophage infiltration, maintaining endothelial barrier function, inhibiting excessive autophagy, and *et al*.^20^,^21^,^24^. Indeed, we observed that the I/R-induced inactivation of Akt was reversed, and I/R-induced inactivation of GSK-3β and mTOR were enhanced in HSPA12B Tg hearts, suggesting that overexpression of HSPA12B activated myocardial PI3K/Akt/mTOR signaling following I/R. Most importantly, administration with PI3K inhibitor Wortmannin abolished the HSPA12B-induced attenuation of cardiac dysfunction provoked by myocardial I/R, suggesting that PI3K/Akt/mTOR signaling plays important roles in mediating the cardioprotection of HSPA12B against I/R injury.

In summary, we provide the first evidence for that HSPA12B overexpression attenuated acute myocardial I/R injury. This protective action of HSPA12B was mediated, at least in part, through activation of PI3K/Akt/mTOR signaling. Targeting HSPA12B expression could be an alternative therapeutic approach for the treatment of myocardial I/R injury.

## Materials and Methods

### Antibodies and Reagents

Wortmannin and Evans blue dye was purchased from Sigma-Aldrich (St Louis, MO). The primary antibody against HSPA12B was a generous gift from Dr. Zhihua Han (East Tennessee State University)^6^. The remaining primary antibodies and the companies that supplied them were as follows: Bcl-2, Bax, Akt and phosphor-Akt, GSK-3β and phosphor-GSK-3β, mTOR and phosphor-mTOR (Cell Signaling Technology, Beverly, MA); PECAM-1 (BD Pharmingen, San Jose, CA); GAPDH (Bioworld Technology, Louis Park, MN); ZO-1 (Invitrogen, Camarillo, CA), Neutrophil (Abcam, Cambridge, MA). The TUNEL assay kit was from Promega (Madison, WI). The supersignal west pico chemiluminescent substrate was obtained from Pierce (Rockford, IL). Trizol reagent was from Life Technology (Carlsbad, CA). FITC-labeled *Lycopersicon esculentum* (Tomato) lectin was from Vector lab (Burlingame, CA).

### Animals

Transgenic mice overexpressing the human *hspa12b* gene driven by its own promoter were developed as described in our previous studies^8^,^12^. Male littermates of HSPA12B Tg and wild type (WT) mice at 8–10 week of age were used in the experiments. Mice were bred and maintained at the Model Animal Research Center of Nanjing University and maintained in the Animal Laboratory Resource Facility at Nanjing University. All experiments were performed according to the guidelines for the “Principles of Laboratory Animal Care” and the “Guide for the Care and Use of Laboratory Animals” published by the NIH (NIH Publication, 8th Edition, 2011). The animal care and experimental protocols were approved by the Nanjing University Committee on Animal Care. All the experiments were conformed to the international guidelines on the ethical use of animals.

### Induction of myocardial I/R injury

Myocardial I/R injury was induced in mice as described previously^32^. Briefly, mice were anaesthetized with inhalation of 1.5–2% isoflurane. The adequacy of anaesthesia was assayed by the disappearance of righting reflex and pedal withdrawal reflex. After anaesthesia, the hearts were exposed and the left anterior descending (LAD) coronary artery was ligated with an 8-0 silk ligature. After occlusion for 45 min, the coronary artery was reperfused by releasing the knot of suture. In sham-operated animals, the same procedure was performed except the LAD ligation. Analgesia was conducted according to our previous studies^12^. For tissue collection, mice were sacrificed by overdose anaesthesia (pentobarbital sodium 150 mg/kg intraperitoneal injection) and cervical dislocation. In PI3K inhibition experiments, mice were treated with a PI3K inhibitor Wortmannin (1mg/kg) by intraperitoneal injection 60 min prior to ischemic insult.

### Determination of myocardial infarct size

Infarct size was examined by TTC staining, as described previously^12^. At 4 h after reperfusion, the hearts were removed and perfused with saline. The hearts were then sliced and incubated in 1.5% TTC for 15 min at 37 °C. Ratios of infarct size vs. ventricle area were measured and expressed as a percentage.

### Echocardiography

Two-dimensional echocardiographic measurements were performed to analyze cardiac function using the Vevo770 system equipped with a 35-MHz transducer (Visualsonics, Toronto, Canada) as our previous methods^8^,^10^. Briefly, Mice were anaesthetized with inhalation of 1.5–2% isoflurane at 24 h after I/R. The measurements were performed by an observer blinded to the treatment. The parameters were obtained in the M-mode tracings at the papillary muscles level and averaged using three to five cardiac cycles.

### Detection of apoptosis in cardiomyocytes

Cardiomyocyte apoptosis in ventricular tissues was examined using the TUNEL assay according to our previously described methods^8^,^10^,^12^. Ventricular tissues at the papillary muscles level were collected 4 h after I/R. The paraffin-sectioning was prepared. Apoptosis in cardiomyocyte was detected using the TUNEL assay. Alpha-actinin was used to stain cardiomyocytes. Hoechst 33342 reagent was used to counterstain the nuclei. Cardiomyocyte apoptosis was observed and counted using a fluorescent microscope (Olympus, Tokyo, Japan) at a magnification of 40× by an observer blinded to the treatment. The data was expressed as the total number of cardiomyocyte apoptosis in each slide view.

### Western blot

Ventricular tissues were collected 4 h after I/R for immunoblotting analysis as described in our previous studies^10^,^12^. Briefly, cellular proteins were prepared, separated on 10% SDS-PAGE, and transferred onto Immobilon-P membranes (Millipore). The membranes were probed with appropriate primary antibodies followed by incubation with peroxidase-conjugated secondary antibodies. The signals were detected by enhanced Pierce chemiluminescence. The blots against GAPDH served as loading controls. The signals were quantified by scanning densitometry and the results from each experimental group were expressed as relative integrated intensity compared with that of controls.

### Immunofluorescence analysis

Ventricular tissues were collected for immunofluorescence staining according to our previous methods^8^. Briefly, ventricular tissues at the papillary muscles level were collected, fixed in 4% paraformaldehyde for 2 h, and incubated with 30% sucrose overnight. The hearts were then frozen in OCT and sectioned at 4 μm. After blocking with 7.5% normal goat serum for 1 h, the cryosections were incubated with the indicated primary antibodies overnight at 4 °C. After thoroughly washing, Cy3- or FITC-conjugated appropriate secondary antibodies were added to the sections to visualize the staining. Hoechst 33342 reagent was used to counterstain the nuclei. The staining was observed using a fluorescence microscope at a magnification of 400× (Olympus, Tokyo, Japan). The fluorescence intensity of ZO-1 was measured more than ten randomly selected areas in each sample using a software (Olympus, Japan). The number of infiltrated neutrophils was counted in more than sixteen randomly areas of each sample.

### Analysis of mRNAs by real time-PCR

Ventricular tissues were collected at 4 h after I/R. Total RNA was extracted and an amount of 2 μg RNA was used for first strand cDNA synthesis by using the oligo (dT) first strand primer. After cDNA synthesis, the expressions of VCAM-1, ICAM-1, VEGF and angiopoietin 1 were estimated by real-time PCR using the SYBR Green Master (Roche, Indianapolis, IN). The PCR results of β-actin served as internal controls. The primers used in the experiments were shown in supplemental information.

### Microvascular leakage

Microvascular leakage was estimated by Evan’s blue dye extravasation according to the previous studies^4^. Briefly, mice were intravenously injected with Evan’s blue dye (3.2 μg/g body weight) at the start of reperfusion after ischemia. After circulation for 30 min, cardiac coronary network was flushed with ice-cold 1% PFA in 0.05M citrate buffer (pH 3.5) via the left ventricle until the perfusion solution was clear from the right atrium. The hearts were then isolated and photographed. For quantification of extravasated Evans blue dye, ventricular tissues was dissolved in formamide (500 μl/mg) on a shaker at 60 °C for 24 h. After centrifugation, the supernatants were read photometrically for the absorbance of Evan’s blue dye color at 610 nm with a Synergy HT plate reader (Bio-Tek, USA). Evan’s blue dye content was expressed as absorbance unites/gram ventricular tissue.

### Myocardial microvascular blood reflow after perfusion

Microvascular blood reflow after perfusion was evaluated by FITC-labeled *Lycopersicon esculentum* (Tomato) lectin (Vector Laboratories) according to the method described previously^4^. Briefly, FITC-labeled Tomato lectin (25 μl) was intravenously injected into mice immediately after reperfusion. Following 30 min reestablishment of circulation, transverse-sectioning at papillary muscles level were prepared and analyzed by fluorescence microscopy.

### Electron microscopy

Ultrathin sections (60–70 nm) were cut with an ultramicrotome^10^. The sections were collected on 200 mesh copper grids (Ernest F. Fullam, Inc.), contrast-stained with uranylacetate and lead citrate, and examined using a JEOL 100-CXtransmission electron microscope.

### Statistical analysis

Results are expressed as mean ± standard deviation (X ± SD). Groups were compared using Student two-tailed unpaired *t* test or one-way analysis of variance analysis (ANOVA) followed by Tukey post hoc test, as appropriate with GraphPad Prism software (GraphPad Software). Statistical significance was set at *P* < 0.05.

## Additional Information

**How to cite this article**: Kong, Q. *et al*. HSPA12B Attenuated Acute Myocardial Ischemia/reperfusion Injury via Maintaining Endothelial Integrity in a PI3K/Akt/mTOR-dependent Mechanism. *Sci. Rep.* **6**, 33636; doi: 10.1038/srep33636 (2016).

## Supplementary Material

Supplementary Information

## References

[b1] Ibanez B., Heusch G., Ovize M. & Van de Werf F. Evolving therapies for myocardial ischemia/reperfusion injury. Journal of the American College of Cardiology 65, 1454–1471, 10.1016/j.jacc.2015.02.032 (2015). 25857912

[b2] Buja L. M. Myocardial ischemia and reperfusion injury. Cardiovasc Pathol 14, 170–175, 10.1016/j.carpath.2005.03.006 (2005). 16009313

[b3] Singhal A. K., Symons J. D., Boudina S., Jaishy B. & Shiu Y. T. Role of Endothelial Cells in Myocardial Ischemia-Reperfusion Injury. Vasc Dis Prev 7, 1–14 (2010). 25558187 10.2174/1874120701007010001 PMC4280830

[b4] Tuuminen R. . Donor simvastatin treatment abolishes rat cardiac allograft ischemia/reperfusion injury and chronic rejection through microvascular protection. Circulation 124, 1138–1150, 10.1161/CIRCULATIONAHA.110.005249 (2011). 21844074

[b5] Eltzschig H. K. & Eckle T. Ischemia and reperfusion–from mechanism to translation. Nat Med 17, 1391–1401, 10.1038/nm.2507 (2011). 22064429 PMC3886192

[b6] Steagall R. J., Rusinol A. E., Truong Q. A. & Han Z. HSPA12B is predominantly expressed in endothelial cells and required for angiogenesis. Arterioscler Thromb Vasc Biol 26, 2012–2018, 10.1161/01.ATV.0000235720.61091.c7 (2006). 16825593

[b7] Hu G. . A novel endothelial-specific heat shock protein HspA12B is required in both zebrafish development and endothelial functions *in vitro*. J Cell Sci 119, 4117–4126, 10.1242/jcs.03179 (2006). 16968741

[b8] Zhou H. . Attenuation of cardiac dysfunction by HSPA12B in endotoxin-induced sepsis in mice through a PI3K-dependent mechanism. Cardiovasc Res 89, 109–118, 10.1093/cvr/cvq268 (2011). 20733008

[b9] Wu J. . HSPA12B inhibits lipopolysaccharide-induced inflammatory response in human umbilical vein endothelial cells. J Cell Mol Med 19, 544–554, 10.1111/jcmm.12464 (2015). 25545050 PMC4369812

[b10] Zhang X. . Involvement of reductive stress in the cardiomyopathy in transgenic mice with cardiac-specific overexpression of heat shock protein 27. Hypertension 55, 1412–1417, 10.1161/HYPERTENSIONAHA.109.147066 (2010). 20439823

[b11] Scarabelli T. M. . Different signaling pathways induce apoptosis in endothelial cells and cardiac myocytes during ischemia/reperfusion injury. Circ Res 90, 745–748 (2002). 11934844 10.1161/01.res.0000015224.07870.9a

[b12] Li J. . HSPA12B attenuates cardiac dysfunction and remodelling after myocardial infarction through an eNOS-dependent mechanism. Cardiovasc Res 99, 674–684, 10.1093/cvr/cvt139 (2013). 23729663

[b13] Reffelmann T. & Kloner R. A. The no-reflow phenomenon: A basic mechanism of myocardial ischemia and reperfusion. Basic research in cardiology 101, 359–372, 10.1007/s00395-006-0615-2 (2006). 16915531

[b14] Schlegel N. & Waschke J. Vasodilator-stimulated phosphoprotein: crucial for activation of Rac1 in endothelial barrier maintenance. Cardiovasc Res 87, 1–3, 10.1093/cvr/cvq093 (2010). 20308204

[b15] Schofield Z. V., Woodruff T. M., Halai R., Wu M. C. & Cooper M. A. Neutrophils–a key component of ischemia-reperfusion injury. Shock 40, 463–470, 10.1097/SHK.0000000000000044 (2013). 24088997

[b16] Liao Y. H. . Interleukin-17A contributes to myocardial ischemia/reperfusion injury by regulating cardiomyocyte apoptosis and neutrophil infiltration. Journal of the American College of Cardiology 59, 420–429, 10.1016/j.jacc.2011.10.863 (2012). 22261166 PMC3262985

[b17] Fernandez-Jimenez R. . Pathophysiology Underlying the Bimodal Edema Phenomenon After Myocardial Ischemia/Reperfusion. Journal of the American College of Cardiology 66, 816–828, 10.1016/j.jacc.2015.06.023 (2015). 26271065

[b18] Bowden R. A. . Role of alpha4 integrin and VCAM-1 in CD18-independent neutrophil migration across mouse cardiac endothelium. Circ Res 90, 562–569 (2002). 11909820 10.1161/01.res.0000013835.53611.97

[b19] Cao Z. . CpG-ODN, the TLR9 agonist, attenuates myocardial ischemia/reperfusion injury: involving activation of PI3K/Akt signaling. Biochim Biophys Acta 1832, 96–104, 10.1016/j.bbadis.2012.08.008 (2013). 22917564 PMC3518630

[b20] Ha T. . Toll-like receptors: new players in myocardial ischemia/reperfusion injury. Antioxid Redox Signal 15, 1875–1893, 10.1089/ars.2010.3723 (2011). 21091074 PMC3159106

[b21] Qi X. F. . Involvement of the FoxO3a pathway in the ischemia/reperfusion injury of cardiac microvascular endothelial cells. Exp Mol Pathol 95, 242–247, 10.1016/j.yexmp.2013.08.003 (2013). 23948278

[b22] Pundir P., MacDonald C. A. & Kulka M. The Novel Receptor C5aR2 Is Required for C5a-Mediated Human Mast Cell Adhesion, Migration, and Proinflammatory Mediator Production. J Immunol 195, 2774–2787, 10.4049/jimmunol.1401348 (2015). 26283482

[b23] De Giusti V. C. . Aldosterone stimulates the cardiac sodium/bicarbonate cotransporter via activation of the g protein-coupled receptor gpr30. J Mol Cell Cardiol 89, 260–267, 10.1016/j.yjmcc.2015.10.024 (2015). 26497404

[b24] Zhai P., Sciarretta S., Galeotti J., Volpe M. & Sadoshima J. Differential roles of GSK-3beta during myocardial ischemia and ischemia/reperfusion. Circ Res 109, 502–511, 10.1161/CIRCRESAHA.111.249532 (2011). 21737790 PMC3158807

[b25] Ma S., Wang Y., Chen Y. & Cao F. The role of the autophagy in myocardial ischemia/reperfusion injury. Biochim Biophys Acta 1852, 271–276, 10.1016/j.bbadis.2014.05.010 (2015). 24859226

[b26] Krug A., Du Mesnil de R. & Korb G. Blood supply of the myocardium after temporary coronary occlusion. Circ Res 19, 57–62 (1966). 5912914 10.1161/01.res.19.1.57

[b27] Bouleti C., Mewton N. & Germain S. The no-reflow phenomenon: State of the art. Arch Cardiovasc Dis 108, 661–674, 10.1016/j.acvd.2015.09.006 (2015). 26616729

[b28] Morishima I. . Angiographic no-reflow phenomenon as a predictor of adverse long-term outcome in patients treated with percutaneous transluminal coronary angioplasty for first acute myocardial infarction. Journal of the American College of Cardiology 36, 1202–1209 (2000). 11028471 10.1016/s0735-1097(00)00865-2

[b29] Heusch G. The Coronary Circulation as a Target of Cardioprotection. Circ Res 118, 1643–1658, 10.1161/CIRCRESAHA.116.308640 (2016). 27174955

[b30] Kohler D. . Phosphorylation of vasodilator-stimulated phosphoprotein prevents platelet-neutrophil complex formation and dampens myocardial ischemia-reperfusion injury. Circulation 123, 2579–2590, 10.1161/CIRCULATIONAHA.110.014555 (2011). 21606399

[b31] Zhang X. . HSPA12B attenuates acute lung injury during endotoxemia in mice. Int Immunopharmacol 29, 599–606, 10.1016/j.intimp.2015.09.022 (2015). 26428851

[b32] Wang X. . MicroRNA-125b protects against myocardial ischaemia/reperfusion injury via targeting p53-mediated apoptotic signalling and TRAF6. Cardiovasc Res 102, 385–395, 10.1093/cvr/cvu044 (2014). 24576954 PMC4030511

