# Supplementary material for: HSPA12B Attenuated Acute Myocardial Ischemia/reperfusion Injury via Maintaining Endothelial Integrity in a PI3K/Akt/mTOR-dependent Mechanism
Source: Sci Rep. 2016 Sep 20;6:33636. doi: 10.1038/srep33636 (PMC5028890; doi:10.1038/srep33636)
Supplement: Supplementary Information [file srep33636-s1.pdf]

HSPA12B Attenuated Acute Myocardial Ischemia/reperfusion Injury via Maintaining Endothelial Integrity in a PI3K/Akt/mTOR-dependent Mechanism

Qiuyue Kong, Leyang Dai, Yana Wang, Xiaojin Zhang, Chuanfu Li, Surong Jiang, Yuehua Li, Zhengnian Ding, Li Liu

Supplemental information

Table 1. Primers used in RT-PCR analysis

HSPA12B	forward	GGCCTGCAAGGGCTGTATATC
	reverse	ATGTGGTTCCAAAGTCGATGG
VCAM-1	forward	AGTTGGGGATTCGGTTGTTCT
	reverse	CCCCTCATTCCTTACCACCC
ICAM-1	forward	GTGATGCTCAGGTATCCATCCA
	reverse	CACAGTTCTCAAAGCACAGCG
Ang-1	forward	GGATGTGCTGTCTAGGCAGAA
	reverse	TTCATGTTCCGGCTTTCCTTT
VEGF	forward	GCCAGACAGGGTTGCCATAC
	reverse	GGAGTGGGATGGATGATGTCAG
ZO-1	forward	CGAGGCATCATCCCAAATAAGAAC
	reverse	TCCAGAAGTCTGCCCGATCAC
β-Actin	forw rd	TAAAGACCTCTATGCCAACACAGT
	reverse	CACGATGGAGGGGCCGGACTCATC

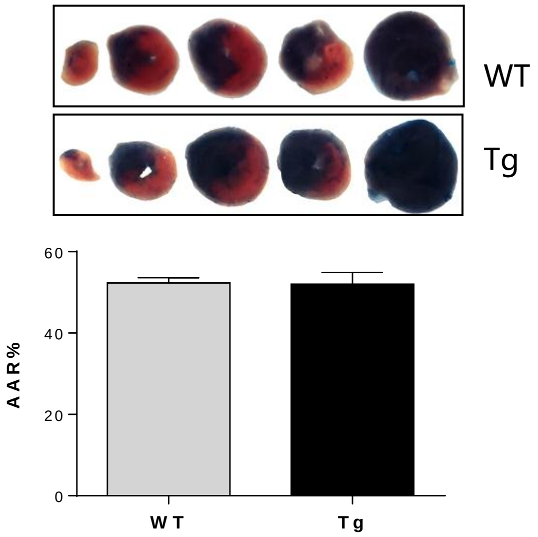

Figure S1. Evaluation of areas at risk of ischemia (AAR).

Mice were perfused intravenously with Evan's blue dye (3.2 μg/ g body weight) before sacrificing. The hearts were isolated and ventricles were sectioned transversally into 5 pieces with equal thickness. The blue-unstained areas (AAR) and blue-stained areas (areas wihtout ischemic risk) were measured using alphaEaseFC software (Informer Technologies, Inc). The total unstained areas and total blue-stained areas of each heart were summed from 5 sections. The AAR was expressed as the percentage of unstained areas to ventricular areas (unstained areas + blue-stained areas). n = 4-5 per group.
